# Precision Approaches to Carbapenem-Resistant Infections in the ICU: Integrating Diagnostics, Stewardship, and Novel Therapies

**DOI:** 10.3390/diagnostics16071053

**Published:** 2026-04-01

**Authors:** Rocco Morena, Sara Palma Gullì, Francesca Serapide, Alessandro Russo

**Affiliations:** 1 Infectious and Tropical Diseases Unit, “Renato Dulbecco” Teaching Hospital of Catanzaro, 88100 Catanzaro, Italy; 2 Department of Medical and Surgical Sciences, “Magna Graecia” University, 88100 Catanzaro, Italy

**Keywords:** carbapenem-resistant infections, intensive care unit, Gram-negative bacteria, antimicrobial stewardship, BL-BLI, CRAB

## Abstract

Carbapenem-resistant Gram-negative infections have become one of the most formidable challenges in intensive care units (ICUs). Critically ill patients—often exposed to invasive procedures, prolonged hospitalization, and broad-spectrum antibiotics—are highly susceptible to infections by carbapenem-resistant Enterobacterales (CRE), *Pseudomonas aeruginosa* (CRPA), and *Acinetobacter baumannii* (CRAB). These pathogens are associated with mortality exceeding 40%, prolonged ICU stays, and increased healthcare costs. Therapeutic advances have reshaped management in recent years. New β-lactam/β-lactamase inhibitor combinations—ceftazidime–avibactam, meropenem–vaborbactam, imipenem–relebactam, and sulbactam–durlobactam—along with cefiderocol, have provided safer and more effective alternatives to previously used regimens. Yet, none are universally effective, particularly against carbapenemase-producing organisms, especially metallo-β-lactamase (MBL) producers, and resistance may still emerge during treatment. Rapid molecular and phenotypic diagnostics, when integrated into antimicrobial stewardship, have improved early therapy alignment and reduced unnecessary broad-spectrum use. Beyond antibiotics, colonization surveillance and infection control remain pivotal, as colonization often precedes invasive infection. Biofilm formation on devices such as endotracheal tubes and catheters further promotes persistence and relapse. Strategies targeting biofilm disruption, improved dosing guided by pharmacokinetic/pharmacodynamic optimization, and therapeutic drug monitoring are crucial in ICU practice. The future of managing these infections will depend on integrating precision tools—rapid diagnostics, mechanism-based therapy, and stewardship-guided decisions—with emerging treatments and adjunctive options such as immunomodulators, bacteriophages, and AI-driven decision support. Continued research in ICU-specific populations, especially regarding pharmacokinetics in patients on ECMO or CRRT, is urgently needed. In summary, while the therapeutic landscape for carbapenem-resistant Gram-negative infections has evolved substantially, sustained success will rely on a multifaceted strategy combining innovation, precision, and prevention to improve outcomes for the most vulnerable patients.

## 1. Introduction

Intensive care units (ICUs) represent the epicenter of nosocomial infections caused by multidrug-resistant (MDR) Gram-negative organisms, due to the lethal combination of highly vulnerable patients and extensively resistant pathogens [1]. Within this group, the pathogens posing the highest danger include carbapenem-resistant *Enterobacterales* (CRE), carbapenem-resistant *Pseudomonas aeruginosa* (CRPA) and carbapenem-resistant *Acinetobacter baumannii* (CRAB), all of which the World Health Organization designates as ‘Critical Priority’ threats [2]. These infections carry major clinical consequences, including few available therapies, protracted ICU admissions and mortality that often exceeds 40% [3]. Historically, treatment relied on polymyxins, tigecycline, fosfomycin and aminoglycosides, despite their limitations related to toxicity and suboptimal pharmacokinetics. The introduction of newer agents—such as ceftazidime–avibactam, meropenem–vaborbactam, imipenem–relebactam, cefiderocol and sulbactam–durlobactam—has expanded therapeutic options; however, resistance and clinical failure remain significant concerns [4,5].

Given the limited number of randomized controlled trials in critically ill populations, several recommendations discussed in this review are based on observational studies, post hoc analyses, or expert guidance, and should be interpreted accordingly.

This review provides an updated overview of the management of carbapenem-resistant infections in intensive care, examining the available evidence, underscoring unresolved challenges and considering emerging directions in antimicrobial innovation and stewardship.

## 2. Epidemiological and Pathophysiological Considerations

### 2.1. Worldwide Dissemination of CRE, CRAB and CRPA

Carbapenem-resistant Gram-negative organisms have become a global healthcare concern, with substantial regional variations in both the prevalence and clinical burden. ATLAS surveillance (2018–2022) reported increasing rates of CRE in Asia Pacific, Latin America, the Middle East–Africa and Europe, whereas North America experienced a slight decline. In contrast, CRPA and CRAB have a high prevalence worldwide, posing a major risk for critically ill patients [6].

A recent global meta-analysis estimated the prevalence of CRPA at 34.7%, with the highest rates in Europe (~47.6%), followed by South America (~40.9%), Africa (~38.5%), North America (~33.3%) and Asia (~32.8%) [7]. Data from Italian ICUs (2021–2023) further illustrate the burden, with CRE and CRPA infection incidences of 3.57 and 1.74 per 1000 patient-days, respectively [8]. In low- and middle-income settings, such as Kenya, the prevalence of carbapenem-resistant Gram-negative bacteria has reached 46.7%, with carbapenem-resistant *Klebsiella pneumoniae* at 35.7%, CRPA at 26.2% and CRAB at 42.9% [9].

### 2.2. Impact on Mortality, Morbidity and Healthcare Costs in the ICU

Infections caused by carbapenem-resistant Gram-negative organisms in the ICU are consistently associated with higher mortality, morbidity and healthcare resource utilization compared with infections due to susceptible strains. Recent multicenter analyses have reported 30-day mortality rates ranging between 35% and 50% in critically ill patients with infections caused by carbapenem-resistant Gram-negative organisms, substantially exceeding those observed in susceptible counterparts [10]. CRAB-related ventilator-associated pneumonia is particularly concerning, with mortality rates frequently reported in the 45–70% range, reflecting the limited effectiveness of available last-line therapies [11,12]. Beyond mortality, these infections contribute to prolonged ICU and hospital stays, increased requirements for invasive procedures and higher rates of complications such as septic shock and multiple organ dysfunction [10]. Economic assessments suggest that carbapenem-resistant infections impose a disproportionate financial burden on healthcare systems, particularly in high-prevalence regions, although estimates of the excess length of stay and per-patient costs vary considerably across settings [10,11].

### 2.3. Molecular Mechanisms of Resistance

Carbapenem resistance in Gram-negative pathogens results from a multifactorial interplay of enzymatic, structural and regulatory mechanisms. The most impactful mechanism is the acquisition of carbapenemases and infections caused by carbapenemase-producing organisms (e.g., KPC-, NDM-, or OXA-producing strains), which efficiently hydrolyze carbapenems and often compromise additional β-lactams. All these types of resistance mechanisms are summarized in Table 1. Importantly, carbapenem resistance is not synonymous with carbapenemase production, as resistance may also arise from non-enzymatic mechanisms such as porin loss and efflux pump overexpression.

### 2.4. Role of Biofilm

Biofilm formation is a key reason carbapenem-resistant organisms persist and spread in the ICU. *Acinetobacter baumannii*, *Pseudomonas aeruginosa*, and certain Enterobacterales can develop thick structured biofilms on endotracheal tubes, catheters, and other medical devices. These communities create a protective environment that reduces antibiotic penetration and shields bacteria from immune defenses. Inside a biofilm, cells adopt different metabolic states and freely exchange resistance genes, allowing them to survive under intense antibiotic pressure. This sessile mode of growth explains why infections often relapse even after apparently adequate therapy. In *A. baumannii*, strong biofilm producers are more likely to persist on hospital surfaces and spread between patients, underscoring their role in ongoing transmission within ICUs [16]. To counter this, researchers are investigating strategies that target biofilm directly, like anti-adhesion surface coatings, quorum-sensing inhibitors that disrupt bacterial communication, and enzymes capable of breaking down the biofilm matrix. Used alongside antibiotics, these approaches could open new paths for preventing and controlling stubborn ICU infections [17].

### 2.5. Predisposing Conditions in Critically Ill Patients

Critically ill patients are highly susceptible to infections caused by carbapenem-resistant Gram-negative pathogens due to prolonged hospitalization, frequent use of invasive devices and extensive exposure to broad-spectrum antimicrobials. Mechanical ventilation compromises natural host defenses and markedly increases the risk of ventilator-associated pneumonia (VAP), a risk further heightened by prior carbapenem therapy, which independently contributes to the development of multidrug-resistant infections in the ICU [18]. Similarly, central venous and urinary catheters provide key portals of entry for bloodstream and urinary tract infections, with observational studies consistently linking their use to CRE and CRAB bacteremia [19]. Moreover, extended ICU length of stay and high severity scores are strongly correlated with colonization and subsequent invasive disease, reflecting both patient frailty and the selective pressure exerted by antimicrobial therapy [20].

Correct and consistent hand hygiene by healthcare workers remains one of the most effective measures to prevent the transmission of carbapenem-resistant organisms in ICUs. Interventions that include hand hygiene training, feedback and promotion of alcohol-based hand rub consumption have been shown to increase compliance rates and reduce healthcare-associated infections [21].

### 2.6. Importance of Rapid Diagnostics for Timely Management

Rapid diagnostic strategies are essential in the ICU to start an appropriate therapy and to optimize outcomes in patients with carbapenem-resistant infections. A 2024 Italian study showed that empiric treatment guided by local surveillance data for pathogens such as *Acinetobacter baumannii*, *Klebsiella pneumoniae* and *Pseudomonas aeruginosa* was associated with improved therapeutic alignment. Early access to susceptibility results, including testing for newer agents such as cefiderocol, also correlated with lower mortality and shorter ICU length of stay [22]. In parallel, a recent open-access review underscored the role of molecular platforms, rapid phenotypic methods and point-of-care assays as pivotal diagnostic innovations. When embedded into diagnostic stewardship frameworks, these tools were shown to facilitate earlier narrowing of antimicrobial coverage and to decrease the frequency of inappropriate empiric regimens [23].

## 3. Clinical Presentation and Diagnostic Modalities

### 3.1. Typical Clinical Manifestations in the ICU

Within intensive care settings, carbapenem-resistant infections most commonly manifest as hospital-acquired or ventilator-associated pneumonia (HAP/VAP). This condition typically emerges 48–72 h after intubation and is defined by the onset of new pulmonary infiltrates, temperature instability, purulent tracheal secretions and progressive impairment of oxygenation [24]. HAP/VAP caused by multidrug-resistant Gram-negative bacteria are associated with higher attributable mortality compared to infections caused by susceptible strains, particularly in regions with a high prevalence of resistance [8].

Bloodstream infections represent another critical presentation, frequently originating from intravascular devices or as secondary complications of respiratory or urinary tract colonization. These episodes are often marked by septic shock, multiple organ dysfunction and extended ICU stays, with mortality rates consistently surpassing those observed in susceptible infections [19]. Complicated urinary tract infections, most often linked to indwelling urinary catheters, are also a significant contributor to morbidity in critically ill populations. In these patients, resistant organisms increase the likelihood of progression from localized infection to pyelonephritis or urosepsis, particularly when initial empiric therapy does not provide adequate coverage [25]. Intra-abdominal infections, such as peritonitis and intra-abdominal abscesses, are being reported with increasing frequency among ICU populations. When caused by CRE or CRAB, these infections are often associated with difficulties in achieving adequate source control, longer durations of hospitalization and higher mortality risk. Recent surveillance data underline the growing involvement of CRE in secondary peritonitis, underscoring the need for timely diagnosis and appropriately tailored empiric regimens [26].

### 3.2. Diagnostic Challenges Due to Coinfections and Colonization

In ICU patients, the frequent coexistence of colonization and coinfections complicates the distinction between true infection and the presence of secondary or colonizing organisms. In suspected VAP, for instance, endotracheal aspirates frequently recover carbapenem-resistant bacteria that may represent colonizers rather than causative agents, while clinical and radiologic findings often lack specificity [27]. Similarly, although CRE carriage is widespread in ICU cohorts, only a subset of colonized individuals develops invasive disease, making positive surveillance cultures useful as risk indicators but insufficient for diagnosis [28]. For *Klebsiella pneumoniae*, recent investigations have shown that reliable discrimination between infection and colonization requires predictive models integrating both clinical features and laboratory data, underscoring the limitations of standard diagnostic methods in this setting [29].

### 3.3. Colonization as a Precursor to Infection and Patient Risk Stratification

Colonization with carbapenem-resistant Gram-negative organisms is often the first step toward invasive infection in ICU patients. Studies suggest that 27.8% of patients colonized with CRE or CRAB eventually develop infection, most often pneumonia or bacteremia, particularly when exposed to invasive devices or broad-spectrum antibiotics [30].

Progression from colonization to infection depends on both host and pathogen factors. Mechanical ventilation, prolonged ICU stay, and mucosal injury—such as from feeding tubes or trauma—allow bacteria to cross into sterile sites. Meanwhile, bacterial traits like biofilm formation, secretion systems, and environmental persistence promote invasion and immune escape. Colonization, therefore, is not a passive state but an active risk phase with clear prognostic and therapeutic relevance [31].

Clinically, identifying and stratifying colonized patients helps target prevention and early treatment. Those with critical illness, immunosuppression, mechanical ventilation, or heavy antibiotic exposure are at highest risk and may benefit from intensified surveillance and tailored empiric regimens. A 2024 cohort found that CRE-colonized patients had a fourfold higher risk of bloodstream infection compared with non-colonized peers [32]. Recognizing colonization early bridges infection control and therapy, paving the way toward truly personalized infection management in critical care.

In addition, coinfections, particularly with fungi or other bacteria, can blur clinical and laboratory signals such as fever or inflammatory markers, leading both to delayed targeted therapy and to unnecessary broad-spectrum antimicrobial use [33].

Diagnostic tools include culture, susceptibility testing, rapid molecular assays and emerging whole-genome sequencing (Table 2).

## 4. Therapeutic Strategies and Current Options

### 4.1. Traditional Agents

Before the introduction of novel β-lactam/β-lactamase inhibitor combinations, management of carbapenem-resistant Gram-negative infections in ICUs relied heavily on so-called “last-line” agents such as colistin, tigecycline and aminoglycosides. While they remain part of the therapeutic armamentarium, their use today is tempered by significant pharmacologic and safety limitations. Regarding colistin (polymyxin E), the current guidance restricts their role in carbapenem-resistant infections: IDSA (2024) discourages polymyxin use when safer effective β-lactam options exist (like for CRE and CRPA) and prefers sulbactam–durlobactam for CRAB, reserving colistin mainly for salvage or when no better alternatives are active [38]. Advantages include retained activity against some multidrug-resistant non-fermenters and a rapid bactericidal effect, which can be relevant in critically ill patients lacking other options. The limitations are substantial: high and early nephrotoxicity in ICU cohorts (often >30–40%) [39], and randomized data show no outcome benefit for adding meropenem to colistin versus colistin alone [40]. Resistance is an ongoing concern, including plasmid-mediated MCR genes (MCR-1 to MCR-10) that can abrogate activity in Enterobacterales, emphasizing stewardship and mechanism-based selection [41].

Tigecycline is considered a second-line option for CRAB and certain CRE infections when other active agents are unavailable or unsuitable [38]. Its main advantages include broad intracellular penetration and utility in mixed intra-abdominal infections. However, limitations include low serum concentrations, making it suboptimal for bacteremia or pneumonia, and gastrointestinal toxicity. Recent studies report emerging resistance, particularly among CRAB isolates, underscoring the need for cautious combination-based use [42,43].

Amikacin remains a valuable adjunctive option against carbapenem-resistant Gram-negative infections when isolates show retained susceptibility. Guidelines recommend its use within combination regimens rather than monotherapy, given rapid resistance selection and nephrotoxicity risks [38]. The international AMINO III study highlighted wide variability in aminoglycoside dosing and monitoring across ICUs, stressing the need for individualized pharmacokinetic-guided therapy to ensure target attainment while minimizing toxicity [44].

Overall, these agents are best considered as reserve therapies, appropriate when susceptibility is confirmed and optimized dosing strategies can be applied, rather than as routine first-line choices in the modern ICU era [38,39,40,41,42,43,44].

### 4.2. Novel β-Lactam/β-Lactamase Inhibitor Combinations

The introduction of novel β-lactam/β-lactamase inhibitor combinations has significantly transformed the therapeutic landscape for carbapenem-resistant infections, especially in ICU patients where the mortality risk is high, and therapeutic options were previously limited to polymyxins, tigecycline, or aminoglycosides. Among the most clinically relevant agents currently available, ceftazidime–avibactam, meropenem–vaborbactam, imipenem–relebactam and ceftolozane–tazobactam are now central to treatment algorithms, each with a distinct activity profile and emerging clinical evidence supporting their use. According to the 2024 IDSA guidelines, ceftazidime–avibactam remains a key first-line therapy for serious infections caused by carbapenemase-producing Enterobacterales, particularly KPC and OXA-48-producing strains. It is approved for use in HAP/VAP as well as bloodstream infections when susceptibility is confirmed [38]. Clinical data consistently show strong efficacy and better safety compared with polymyxin-based regimens, especially in critically ill patients [45]. The main drawback of ceftazidime–avibactam is its lack of activity against MBL producers such as NDM, VIM, and IMP. Resistance can also develop during prolonged therapy or in infections with high bacterial loads, often linked to blaKPC-3 mutations or porin alterations [46]. Encouragingly, global surveillance data from the ATLAS program (2018–2022) report resistance rates under 5% among KPC-producing *Klebsiella pneumoniae*, reinforcing ceftazidime–avibactam’s role as a cornerstone of mechanism-guided therapy [6].

Meropenem–vaborbactam is a β-lactam/β-lactamase inhibitor combination active against KPC-producing Enterobacterales but inactive against MBLs or OXA-48-like enzymes. Guidelines recommend it as a preferred therapy for severe CRE infections, particularly bloodstream and urinary tract infections, when susceptibility is confirmed [38]. The TANGO I randomized trial demonstrated noninferiority to piperacillin–tazobactam for complicated UTIs, with excellent safety [47], while TANGO II showed significantly higher clinical cure and lower nephrotoxicity versus best available therapy in serious KPC-CRE infections [48]. Limitations are the lack of activity against MBL or OXA-48-like enzymes and the risk of resistance via permeability changes. Emerging resistance has been documented through porin (ompK36/ompK35) mutations coupled with increased blaKPC copy number [46].

Imipenem–relebactam combines a carbapenem with a diazabicyclooctane β-lactamase inhibitor active against class A (KPC) and class C (AmpC) enzymes but not MBL or OXA-48-like variants. Current IDSA 2024 and ESCMID 2023 guidelines recommend it for severe infections caused by KPC-producing Enterobacterales and difficult-to-treat *Pseudomonas aeruginosa*, particularly when alternative β-lactam/β-lactamase inhibitor combinations are unsuitable [38]. The RESTORE-IMI 1 trial showed that imipenem–relebactam achieved similar clinical cure with less nephrotoxicity versus colistin plus imipenem in carbapenem-resistant infections [49], while RESTORE-IMI 2 confirmed non-inferiority to piperacillin–tazobactam in hospital-acquired and ventilator-associated pneumonia [50]. Its main advantages include safety and reliable activity against AmpC and KPC mechanisms; still, resistance can arise through porin mutations or blaKPC variants [46].

Ceftolozane–tazobactam is a β-lactam/β-lactamase inhibitor combination recommended by IDSA 2024 for infections caused by *Pseudomonas aeruginosa* resistant to standard antipseudomonal agents but not producing carbapenemases [38]. Its main advantage lies in its strong activity against *Pseudomonas aeruginosa* with AmpC overexpression or porin loss, making it valuable for HAP/VAP and complicated intra-abdominal infections [51]. Limitations include its inactivity against MBL and OXA-type producers and reduced efficacy against most CRE. Resistance can develop through ampC mutations or efflux pump upregulation, highlighting the need for targeted use and stewardship [52].

Infections caused by carbapenemase-producing Enterobacterales, particularly MBL-producing strains, remain among the most difficult to treat in the ICU, since none of the available novel β-lactam/β-lactamase inhibitor combinations are active against these enzymes. The combination of ceftazidime–avibactam with aztreonam represents one of the most effective current strategies for treating infections due to this kind of bacteria, including NDM, VIM, and IMP producers. According to IDSA 2024 guidance, this combination should be considered when MBLs are detected, as avibactam inhibits co-produced serine β-lactamases while aztreonam remains stable to MBL hydrolysis [38]. Clinical data demonstrate high clinical success rates and improved survival compared with older polymyxin-based regimens [53]. Its advantages include the broad coverage of dual enzyme mechanisms and favorable safety. Limitations are the need for co-administration, the dosing complexity, and limited randomized data. Emerging resistance has been linked to mutations in ampC or blaNDM genes [54].

Taken together, these novel β-lactam/β-lactamase inhibitor combinations have reshaped the management of carbapenem-resistant Gram-negative infections in the ICU. Their utility is maximized when treatment decisions are guided by rapid diagnostics that clarify the underlying resistance mechanism, since their spectra differ significantly. Ceftazidime–avibactam is currently the most versatile for Enterobacterales, with coverage of both KPC and OXA-48-like producers, though it requires combination with aztreonam when MBLs are present. Meropenem–vaborbactam and imipenem–relebactam are particularly useful in KPC-endemic settings and provide safer more effective alternatives to colistin-based regimens. For *Pseudomonas* aeruginosa, imipenem–relebactam holds a distinct advantage in cases with specific resistance mechanisms. In the ICU, where infection onset is rapid, and outcomes are tightly linked to early administration of active therapy, these agents have provided not only new therapeutic efficacy but also the possibility to reduce reliance on toxic legacy drugs, aligning patient safety with antimicrobial stewardship goals [45,46,47,48,49,50,51,52,53,54].

### 4.3. Innovative Option

The activity of newer agents against carbapenem-resistant pathogens depends on the underlying resistance mechanisms. While β-lactam/β-lactamase inhibitor combinations primarily target carbapenemase-producing organisms with serine enzymes (e.g., KPC, OXA) and lack activity against MBLs, cefiderocol is a siderophore cephalosporin that exploits iron transport systems for cell entry and remains stable to hydrolysis by most β-lactamases, including MBLs, while being less affected by porin loss and efflux mechanisms [54]. Both IDSA (2024) and ESCMID (2023) guidelines recommend it as a preferred or alternative therapy for severe infections caused by CRE and CRAB, particularly when other β-lactams are inactive or unavailable [38,55]. The CREDIBLE-CR phase 3 trial demonstrated cefiderocol’s efficacy across diverse carbapenem-resistant Gram-negative pathogens, though higher mortality was observed in the *Acinetobacter* subgroup, likely reflecting disease severity rather than reduced drug activity [56]. The APEKS-NP trial subsequently confirmed non-inferiority to meropenem in nosocomial pneumonia, including cases due to multidrug-resistant Gram-negatives [57]. Clinical experience supports its use in bloodstream and respiratory infections caused by CRE, with observational studies reporting favorable outcomes when used early and guided by susceptibility testing [58]. Advantages include potent in vitro activity against most carbapenemase producers, predictable pharmacokinetics, and suitability for monotherapy in susceptible isolates. Limitations include limited data for CRAB infections, potential for heteroresistance, and decreased efficacy in isolates with permeability or efflux mutations [59]. Emerging resistance, though rare, has been associated with mutations in siderophore receptor genes (pirA, cirA) or β-lactamase upregulation after exposure, underscoring the need for vigilance and stewardship [60].

Eravacycline is a fully synthetic fluorocycline related to tigecycline but designed to overcome common tetracycline resistance mechanisms such as efflux pumps and ribosomal protection. It shows broad activity against multidrug-resistant Gram-negative bacteria, particularly *Acinetobacter baumannii* and some CRE, though it lacks efficacy against *Pseudomonas* aeruginosa [61]. Both the 2024 IDSA and 2023 ESCMID guidelines list eravacycline as an alternative for complicated intra-abdominal infections (cIAI) caused by resistant organisms when β-lactam options are limited [38,62]. Phase III trials (IGNITE 1 and 4) demonstrated non-inferiority to ertapenem and meropenem in cIAI, with good tolerability and few gastrointestinal side effects [63,64]. Real-world studies report over 70% success in treating CRAB infections [65]. With once-daily dosing and strong tissue penetration, eravacycline is a valuable option for MDR Gram-negative infections, mainly in intra-abdominal or soft-tissue settings [66]. Emerging resistance, primarily linked to tet(X) gene variants, has been reported among clinical *Acinetobacter* isolates, warranting continued surveillance [67].

Aztreonam/avibactam is a recently developed fixed-dose combination that pairs the well-established antibiotic aztreonam with the β-lactamase inhibitor avibactam. This formulation has demonstrated both in vitro and in vivo efficacy against carbapenem-resistant Enterobacterales, including strains producing extended-spectrum β-lactamases (ESBLs), serine carbapenemases, and MBLs [68].

Furthermore, two prospective observational studies reported a clinical benefit from maintaining aztreonam activity by administering it alongside ceftazidime–avibactam (both given every eight hours), compared with alternative antimicrobial therapies, including colistin-based regimens, in severe infections caused by MBL-producing Enterobacterales [53,69]. Nevertheless, dosing strategies based on this empirical combination approach may not always be optimal.

Plazomicin is a next-generation aminoglycoside engineered to evade most aminoglycoside-modifying enzymes while retaining potent activity against CRE, including KPC- and OXA-48-producing strains [70]. According to IDSA 2024 guidelines, plazomicin is considered an alternative option for bloodstream and urinary tract infections caused by susceptible CRE when β-lactam agents are unsuitable or resistance mechanisms are unclear [38]. The EPIC phase III trial demonstrated the non-inferiority of plazomicin to meropenem for complicated UTIs, while the CARE trial showed lower mortality versus colistin in CRE bacteremia, highlighting its clinical value and improved safety profile [70,71]. Nevertheless, plazomicin lacks activity against *Pseudomonas aeruginosa* and *Acinetobacter baumannii*, and resistance can emerge via 16S rRNA methyltransferases, emphasizing the importance of molecular resistance screening [72].

Sulbactam–durlobactam is a newly approved β-lactam/β-lactamase inhibitor combination specifically designed to treat infections caused by CRAB [73]. The IDSA 2024 guidelines recommend sulbactam–durlobactam as a preferred therapy for serious CRAB infections, including hospital- and ventilator-associated pneumonia, when susceptibility is confirmed [38]. The pivotal ATTACK phase 3 trial showed that sulbactam–durlobactam was non-inferior to colistin in 28-day all-cause mortality and achieved similar or higher clinical cure rates with significantly lower nephrotoxicity (19% vs. 32%) [74]. Resistance remains uncommon but has been linked to mutations in blaOXA-23 or PBP3 targets, highlighting the need for molecular surveillance [75,76,77].

Real-world ICU data remain limited, but early experience and post-approval analyses suggest favorable clinical response rates and a substantially improved safety profile compared with colistin-based regimens, including in severe pneumonia and ventilator-associated infections [74,78]. Sulbactam–durlobactam offers targeted activity against OXA-producing *A. baumannii* through the inhibition of class D β-lactamases, providing a mechanistic advantage in high-burden infections while significantly reducing the risk of nephrotoxicity associated with polymyxins [78,79]. Resistance appears uncommon but has been linked to specific genetic backgrounds, particularly mutations affecting PBP3 or the presence of MBL, underscoring the need for susceptibility testing and ongoing surveillance during prolonged therapy [75].

### 4.4. Pipeline Agents in Late-Stage Development

Beyond currently available options, several promising agents are progressing through Phase II and III trials against carbapenem-resistant Gram-negative pathogens, particularly *Acinetobacter baumannii* complex and Enterobacterales. In addition to cefiderocol and sulbactam–durlobactam (already approved for CRAB), four novel compounds are under investigation: BV-100, cefepime–zidebactam, zosurabalpin and OMN6. Each offers a different strategy; cefepime–zidebactam combines a known cephalosporin with a potent inhibitor, while OMN6 (an antimicrobial peptide) uses an entirely new approach based on membrane disruption, without promoting antimicrobial resistance. Such diversity is essential as existing choices continue to narrow [80].

Zosurabalpin, in particular, has drawn significant attention: early data show strong in vitro activity against CRAB, preparing Phase III studies in pneumonia and sepsis caused by *Acinetobacter* spp. If successful, it could become the first late-stage drug specifically targeting CRAB [81].

Another important candidate is taniborbactam, a bicyclic boronate inhibitor with broad β-lactamase coverage, including some MBL, when paired with the right β-lactams. It is considered one of the more advanced programs in the antibiotic resistance pipeline [82].

Overall, although the antibiotic pipeline remains thin compared with clinical needs, these agents represent much-needed hope. Their impact will depend on ongoing randomized controlled trials that test not only laboratory potency but also clinical efficacy, safety, pharmacokinetics in critically ill patients and the risk of resistance development in ICU settings.

### 4.5. Non-Conventional Therapies

Beyond conventional antibiotics, non-traditional strategies are being investigated for carbapenem-resistant infections. Bacteriophage therapy has attracted growing interest as a potential adjunctive strategy for severe infections caused by carbapenem-resistant Gram-negative pathogens, particularly when conventional options are limited or poorly tolerated. Early clinical experiences, including compassionate-use cases and small case series, suggest that phages may help reduce the bacterial burden and support infection control in selected multidrug-resistant infections [83,84]. However, the available evidence remains limited and heterogeneous, with important unresolved issues concerning regulatory pathways, individualized phage selection, formulation, timing of administration, and the lack of standardized treatment protocols. At present, phage therapy should still be considered an investigational and highly specialized approach [83,84]. In parallel, experimental antibiotic combinations have shown synergy in vitro, for example cefepime with amikacin or cefepime with ampicillin–sulbactam, occasionally restoring activity against extensively drug-resistant *Acinetobacter baumannii* [85]. These strategies highlight the potential of alternative or complementary mechanisms, but their role in ICU care is far from defined. Rigorous clinical studies are needed to confirm their efficacy, optimize the dosing and understand pharmacokinetics in critically ill patients, while also monitoring the risk of resistance.

### 4.6. Combination Therapy vs. Monotherapy

The optimal therapeutic strategy for carbapenem-resistant Gram-negative infections—whether monotherapy or combination regimens—remains one of the most debated topics in critical care. IDSA guidance generally discourages routine combination therapy. In the ICU, where patients often experience septic shock, organ failure, and altered pharmacokinetics, the choice can profoundly affect the outcomes. Recent meta-analyses indicate that combination therapy may provide a modest but consistent survival advantage compared to monotherapy, especially in infections caused by highly resistant pathogens [86]. Still, the current evidence is largely derived from observational studies and meta-analyses, with limited randomized data for novel β-lactam/β-lactamase inhibitor regimens.

For CRE, the evidence suggests that combination therapy—most often pairing a novel β-lactam/β-lactamase inhibitor (such as ceftazidime-avibactam or meropenem-vaborbactam) with an aminoglycoside or fosfomycin—can reduce microbiological failure and mortality in critically ill patients with bloodstream infections [38]. Observational cohorts have shown that dual therapy may be particularly beneficial in severe infections with high bacterial load or when the pathogen exhibits borderline susceptibility, enhancing bacterial clearance and reducing resistance emergence [87]. However, when pathogens are fully susceptible to new β-lactam/β-lactamase inhibitors combinations, monotherapy with these agents has achieved comparable outcomes, supporting their use as single-drug therapy in stable patients with confirmed susceptibility [88].

In contrast, CRAB presents a more complex challenge. The pathogen’s multifactorial resistance mechanisms and biofilm formation make treatment less predictable. Studies evaluating colistin-based combinations have shown mixed results, often limited by toxicity and lack of consistent benefit [89]. The approval of sulbactam–durlobactam has changed the therapeutic paradigm, offering a rational backbone for combination strategies. Early evidence suggests that adding agents such as meropenem or ampicillin–sulbactam may improve bacterial eradication and prevent resistance selection in high-inoculum infections [90]. Nonetheless, the incremental benefit over optimized monotherapy remains to be validated in larger randomized ICU trials.

In CRAB, the rationale for combination therapy is often strongest when infection biology makes eradication difficult, especially in VAP and device-related infections, where endotracheal-tube biofilms can act as a persistent reservoir linked to incomplete response and relapse. Biofilm growth limits antibiotic penetration and promotes phenotypic tolerance/persistence; so, “susceptible” MICs may still translate into slow or incomplete killing in vivo [16]. In addition, heteroresistance to last-line agents (notably colistin and tigecycline) has been documented in clinical *A. baumannii* isolates, providing a biological basis for using two active drugs in high-burden disease [91]. Early source control, including device removal or replacement when feasible, remains essential [92].

Ultimately, the decision between monotherapy and combination therapy in critically ill patients should be guided by mechanism-based susceptibility testing, infection site, and host factors. While combination regimens remain justified for the most resistant CRAB and selected CRE cases, excessive or empiric combination use should be avoided to limit toxicity and preserve future treatment options [93].

## 5. Role of Therapeutic Drug Monitoring (TDM)

Therapeutic Drug Monitoring (TDM) is increasingly vital for optimizing antibiotic use in critically ill patients, particularly those with multidrug- or carbapenem-resistant infections. Profound pharmacokinetic variability in the ICU—driven by altered perfusion, renal clearance, and extracorporeal support—makes drug exposure unpredictable. TDM offers a precision approach, adjusting doses to maintain pharmacodynamic targets such as time above MIC for β-lactams. A 2024 meta-analysis showed that β-lactam TDM improves clinical cure and bacterial eradication in ICU patients [94], while prospective studies confirm better target attainment and possibly lower mortality in severe sepsis [95]. Expert guidelines now advocate extending TDM to polymyxins and aminoglycosides as part of antimicrobial stewardship programs [96]. Despite strong evidence, implementation remains uneven due to limited rapid assays and trained staff. Wider use of real-time or bedside TDM, supported by pharmacometric modelling, could significantly enhance treatment precision and outcomes in carbapenem-resistant infections.

## 6. Infection Control and Antimicrobial Stewardship in the ICU

Effective control of CRE, CRAB and CRPA in ICUs rests upon a dual approach: infection prevention and control (IPC) strategies and antimicrobial stewardship (AMS) programs. From an IPC perspective, rigorous hand hygiene, contact precautions, patient cohorting or isolation and environmental cleaning are foundational [21]. In one ICU outbreak of CRAB, a five-component bundle (reinforcement of hand hygiene, screening cultures, contact precautions, environmental sampling and disinfection) ultimately reduced ICU-CRAB incidence from ~30 per 1000 patient-days to zero over time, supporting the power of bundled interventions [97]. AMS programs complement IPC by reducing selective antibiotic pressure, especially from carbapenems and broad-spectrum agents. A study in a tertiary ICU showed that combining AMS programs initiatives (such as carbapenem-sparing policies) and IPC efforts led to decreased carbapenem use, lower resistance rates in *Acinetobacter baumannii* and reduced colonization pressure. Real-world data underline that stewardship interventions can produce durable reductions in resistance when tied with IPC [98]. Active surveillance cultures (screening upon ICU admission and periodically) help identify colonized patients early, allowing targeted isolation and monitoring. In ventilator-associated settings, screened positive patients with CRE/CRAB have a much higher risk of progression to invasive infection, and negative screening offers useful negative predictive value to guide de-escalation. Environmental control is equally critical: while enhanced cleaning alone may not always sufficient—one study found no significant reduction in CRAB acquisition by cleaning alone in ICU—integrated bundles combining cleaning, disinfection and workflow changes are more successful [99,100]. In ICU settings, measures must be tailored: cohort staffing (dedicated personnel to colonized/infected patients), surveillance of high-touch surfaces, avoiding shared devices and rapid communication between microbiology, AMS and ICU teams are crucial. In practical ICU settings, antimicrobial stewardship should be operationalized through a set of key actions:

- Reassessment of antimicrobial therapy within 48–72 h based on clinical evolution and microbiological data;
- Avoidance of antibiotic treatment in cases of colonization without evidence of infection;
- Use of mechanism-based therapy guided by rapid diagnostics and local epidemiology;
- Early de-escalation driven by antimicrobial susceptibility testing (AST) results.

When consistently applied, these actions improve early appropriateness of therapy and reduce unnecessary antibiotic exposure in critically ill patients.

## 7. Prognostic Implications and Outcomes

Outcomes in ICU patients with CRE/CRAB/CRPA are driven by both pathogen and host factors. Across large cohorts, the severity of illness at presentation (shock, respiratory failure, acute kidney injury), high bacteremia scores and lack of source control consistently predict death. In a national cohort of hospitalized patients with *Acinetobacter baumannii*, acute respiratory failure, shock and acute renal failure independently predicted mortality, underscoring how organ dysfunction dominates prognosis in this population [101]. In HAP/VAP due to CRAB, the 21-day mortality and ventilator dependence were high; older age, higher SOFA and vasopressor use were independent risk factors, highlighting the compounded risk created by critical illness severity and hemodynamic instability [102]. For CRE bloodstream infection, a multicenter analysis reported ~42% 30-day mortality, with the Pitt bacteremia score, immunocompromise, no source control and inappropriate empirical therapy as independent predictors [103]. These data align with broader observational evidence showing higher mortality for CRAB infections vs. susceptible infections, particularly in severe pneumonia and bloodstream infections [104]. The timeliness of effective therapy is among the few modifiable determinants of outcome. The ECDC’s 2025 risk assessment concludes that the high attributable mortality in CRE is primarily due to delays in administration of effective antimicrobial therapy and the limited number of alternative treatment options” [105]. Beyond guidance, recent clinical studies show outcome gains when active therapy is started early and then targeted to the organism and mechanism. In Enterobacterales bloodstream infections, receiving early phenotype-desirable antimicrobial therapy (i.e., the narrowest active β-lactam for the phenotype) was associated with better 30-day outcomes, supporting prompt optimization once diagnostic data are available [106]. In settings with mixed NDM/OXA-48 epidemiology, sepsis/septic shock and neutropenia independently increased early mortality in CRE bloodstream infections, but specific active regimens (for example fosfomycin-based combinations) were linked with improved survival, illustrating how mechanism-directed choices can modify the risk in high-severity presentations [107]. Collectively, these findings argue for accelerated diagnostics, aggressive PK/PD optimization (prolonged infusions) and rapid de-escalation or escalation to mechanism-active agents as soon as the data permit. Having multiple comorbidities not only raises the chance of treatment failure but also increases the risks of therapy itself. Conditions like chronic kidney disease, cancer, or immunosuppression reduce physiologic reserves and complicate dosing. Support devices such as CRRT or ECMO, along with fluctuating renal function, further change drug exposure and can delay reaching PK/PD targets. In *Acinetobacter baumannii* infections, higher comorbidity burden and clinical instability are linked with poorer outcomes [94], while in CRAB pneumonia, respiratory severity and hemodynamic compromise strongly predict mortality and ventilator dependence [102]. For CRE bloodstream infections, host factors—including immunosuppression and bacteremia severity—remain independent drivers of outcome, even after adjusting for the treatment received [103,107]. These realities call for individualized management: rapid source control, timely initiation of active therapy, dose adjustment supported by therapeutic drug monitoring when available and ongoing reassessment as organ function changes. Ultimately, prognosis depends as much on the patient’s baseline status as on how quickly and precisely clinicians deliver effective therapy and source control.

More advanced ICU-specific strategies remain debated but potentially relevant. Selective digestive decontamination (SDD) and routine chlorhexidine bathing have shown variable benefits and raise ecological concerns in CRE/CRAB-endemic settings [108]. From a stewardship perspective, emerging tools include AI-based decision support systems for early targeted therapy and model-informed precision dosing platforms to optimize PK/PD exposure in critically ill patients [109].

## 8. Future Directions and Emerging Therapies

The management of antimicrobial resistance in the ICU remains challenging. Looking ahead, three areas stand out as the next frontier: new antibiotics in the pipeline, adjuvant and immunomodulatory approaches and the growing role of AI in precision therapy. Several agents are now moving into clinical evaluation.

One of the most advanced is cefepime–zidebactam (WCK 5222). Zidebactam not only boosts cefepime’s activity as a β-lactam enhancer but also binds PBP2, restoring potency against resistant Enterobacterales, *Pseudomonas aeruginosa* and even some *Acinetobacter baumannii* isolates. Early in vitro work and limited clinical reports suggest strong activity, including against NDM-producing *Pseudomonas* in invasive disease [80].

Another candidate, taniborbactam (VNRX-5133), is a cyclic boronate inhibitor with the rare ability to block both serine β-lactamases and some MBL. Paired with cefepime, it has already shown efficacy in complicated urinary tract infection trials, though regulatory hurdles remain [82]. Together, these agents highlight the next generation of β-lactam/β-lactamase inhibitor therapies designed to reach beyond the limits of older inhibitors.

Severe infections in the ICU are not just a matter of unchecked bacterial growth; they also reflect a deep imbalance in the host immune system. This has sparked growing interest in combining antibiotics with immunomodulatory therapies to improve patient outcomes. Several strategies are being investigated: monoclonal antibodies directed against bacterial surface antigens or toxins, such as anti-endotoxin mAbs, are designed to neutralize lipopolysaccharide and dampen the harmful inflammatory cascade. Other approaches aim at cytokine modulation, seeking to restore innate immune responses that are blunted during sepsis. In experimental models of Gram-negative pneumonia and bacteremia, adding these interventions to antibiotics has led to better bacterial clearance and improved survival [110].

Beyond pathogen-targeted approaches, therapies that recalibrate host immunity are also gaining momentum. Immune checkpoint inhibitors like anti-PD-1 may help reverse sepsis-induced immunoparalysis. IgM-enriched immunoglobulin preparations are under study as a way to strengthen humoral defenses. Even cell-based therapies, such as mesenchymal stem cells, are being explored for their ability to reset the immune balance [111]. Clinical evidence so far is limited and often inconsistent, yet emerging reviews suggest that precision immunotherapy, guided by biomarkers and patient endotypes, may offer a path to real impact.

In the ICU, integrating these approaches with optimized antibiotic regimens could represent a future step toward truly personalized care. ICU care is becoming increasingly data-driven. Artificial intelligence (AI) is beginning to support antibiotic choices in real time. A 2025 pilot study tested an AI-based decision support system in sepsis, showing that it could integrate patient factors, labs and microbial risk to recommend early targeted regimens [112]. At the same time, precision medicine models are being adapted to predict who will respond to which antibiotic, incorporating prior microbiology, PK/PD data and local resistance patterns. Linking these tools with electronic health records and rapid diagnostics could eventually allow dynamic individualized prescribing, moving clinicians away from broad empiricism and helping contain resistance.

## 9. Conclusions

Over the past decade, ICUs have made real progress in treating carbapenem-resistant Gram-negative infections. New drugs such as ceftazidime–avibactam, meropenem–vaborbactam, imipenem–relebactam, cefiderocol and sulbactam–durlobactam have replaced older toxic regimens with safer and more effective options, yet none of these offers a panacea. Real-world experience has shown these agents can improve outcomes in bloodstream infections, ventilator-associated pneumonia and intra-abdominal infections, but resistance can emerge quickly and must be watched closely [42,43,44,45,46,47,48,49,50,51,52,53,54,55,56,57,58,59,60,61,62,63,64,65,66,67,68,69,70,71,72,73,74,75,76,77,113]. From a clinical perspective, a few priority principles can guide management in ICU settings:

- Ensure early appropriate therapy and adopt a mechanism-based approach whenever possible, pairing rapid diagnostics with targeted therapy to minimize empiricism and avoid unnecessary broad-spectrum exposure [22,23].
- Consider local epidemiology for better empirical strategies.
- Use optimized PK/PD dosing strategies (extended or continuous infusions, TDM where available), especially in unstable critically ill patients whose pharmacokinetics deviate significantly.
- Reserve combination therapy for the sickest patients, forced scenarios lacking a single active agent, or where resistance suppression is critical, while carefully weighing the toxicity risks.
- Prioritize infection control and stewardship integration in ICU workflows: daily antibiotic review, de-escalation, cohorting, environmental control and surveillance help preserve the efficacy of new antibiotics [97,98,99,100].
- Monitor closely for on-therapy resistance, especially when using newer agents and consider fallback or salvage options early.

These principles help translate complexity into actionable decisions in critically ill patients. The road ahead is equally demanding. Randomized controlled trials in ICU populations are still too few, especially the ones about CRAB and trials comparing monotherapy and combination regimens with modern drugs. We also need more pharmacokinetic data in special groups—patients on ECMO or CRRT, the obese, children. Faster phenotypic resistance tests must be paired with stewardship to shorten the empirical window. New therapeutics, from novel β-lactam/β-lactamase inhibitor agents to immunomodulators and bacteriophage therapy, need rigorous evaluation in the ICU setting, where organ dysfunction and comorbidity complicate translation. Finally, artificial intelligence could soon help us move from empiric to precise therapy, optimize dosing and even anticipate resistance.

In short, the therapeutic landscape has matured, but lasting success depends on integration: better drugs, epidemiological knowledge, smarter diagnostics, stronger stewardship and adaptive research, all working together to protect the sickest patients [114,115,116,117,118,119].

**Figure 1:**
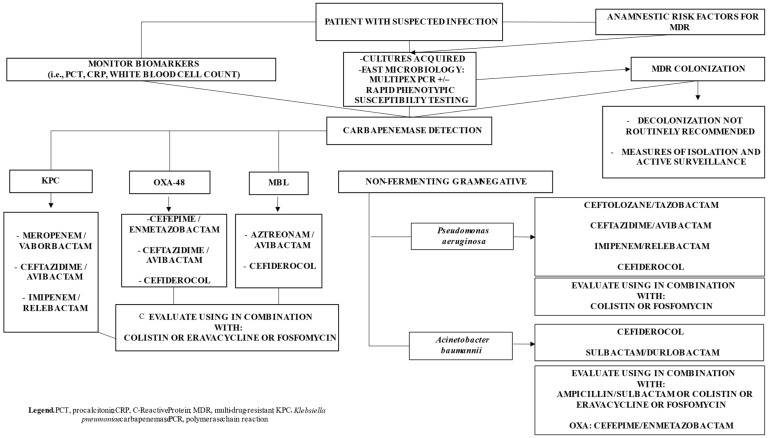
Simplified diagnostic and therapeutic framework for carbapenem-resistant Gram-negative infections in critically ill patients.

A simplified therapeutic framework for the management of carbapenem-resistant Gram-negative infections in critically ill patients is shown in Figure 1. The figure summarizes a stepwise approach from clinical suspicion and risk stratification to early diagnostics, targeted therapy, reassessment, and stewardship integration.

## Figures and Tables

**Table 1:** Main molecular mechanisms of carbapenem resistance in key Gram-negative pathogens.

Pathogen	Primary Carbapenemase(s)	Additional Mechanisms	Functional Effect	Clinical Implication/ICU Relevance
*Klebsiella pneumoniae*	KPC, NDM, OXA-48-like	Overexpression of AcrAB-TolC efflux system; loss or modification of porins (OmpK35, OmpK36)	Enhanced drug extrusion and reduced intracellular antibiotic concentration	High-level resistance to carbapenems and other β-lactams; frequent cause of bloodstream infections and outbreaks in ICUs [13]
*Acinetobacter baumannii*	OXA-23, OXA-24/40, OXA-58	Porin downregulation (loss of CarO); overexpression of AdeABC and AdeIJK efflux pumps	Decreased permeability and increased efflux leading to very high MICs	Major cause of ventilator-associated pneumonia and bacteremia; persistence in hospital environments facilitates ICU transmission [14]
*Pseudomonas aeruginosa*	VIM, IMP, NDM (MBLs)	Loss or alteration of OprD porin; overexpression of MexAB-OprM and MexXY-OprM efflux systems	Reduced carbapenem uptake and active extrusion; frequent coexistence with metallo-β-lactamases	Causes multidrug- and extensively drug-resistant infections, particularly HAP/VAP and sepsis in critically ill patients [15]
*Escherichia coli*	NDM, KPC, OXA-48-like	Downregulation or loss of OmpC and OmpF porins; co-expression of AcrAB-TolC efflux pump	Reduced outer membrane permeability and increased efflux; synergistic effect with carbapenemases	Common source of CRE urinary tract and bloodstream infections; potential for plasmid-mediated spread of carbapenemase genes across Enterobacterales [13]

Note: Carbapenem resistance in *E. coli* is typically plasmid-mediated but frequently enhanced by permeability defects and efflux pump overexpression. The coexistence of multiple mechanisms leads to highly resistant phenotypes and limits treatment options, particularly in ICU patients with device-associated infections. **Abbreviations and Acronyms**: **KPC**—*Klebsiella pneumoniae* carbapenemase (Class A β-lactamase); **NDM**—*New Delhi metallo-β-lactamase* (Class B); **OXA**—Oxacillinase (Class D β-lactamase); **VIM**—*Verona integron-encoded metallo-β-lactamase* (Class B); **IMP**—*Imipenemase* (Class B).

**Table 2:** Diagnostic tools for CRE detection in ICU settings.

Diagnostic Tool	Description/Use	Turnaround Time	Advantages	Limitations
Traditional culture and AST	Cornerstone for pathogen identification and antimicrobial susceptibility testing.	48–72 h	Reliable; provides phenotypic susceptibility data.	Long turnaround time; delays targeted therapy in critically ill patients [34].
Rapid AST	Methods based on cell growth or morphology (real-time growth observation); cell metabolism (measurement of metabolic activity); rapid genotyping (identification of known resistance genes)	2–6 h	Faster results for targeted treatment as soon as possible; better management of serious infections (e.g., sepsis); reduced empirical use of broad-spectrum antibiotics; fight against global antibiotic resistance.	Limited availability; variability in performance across platforms; still requires viable isolates [35].
Rapid molecular/immunochromatographic assays (NG-Test^®^ CARBA 5)	Detect major carbapenemase families directly from blood culture broth.	~30–60 min	Rapid results: reduces diagnostic delay compared with conventional culture.	Detect only predefined carbapenemase genes; do not identify non-enzymatic resistance mechanisms (e.g., porin loss, efflux pumps) [34].
Multiplex PCR panels	Detect multiple resistance genes simultaneously.	1–2 h	Shorten time to targeted therapy; minimize unnecessary broad-spectrum antibiotic use.	Restricted gene panels; inability to detect unknown or uncommon resistance mechanisms; lack of phenotypic susceptibility data [36].
Whole-genome sequencing (WGS)	Characterizes CRE isolates from bloodstream infections; identifies resistance determinants and clonal spread.	1–2 days (post-isolation)	High-resolution data; supports outbreak investigation and infection control.	Requires specialized infrastructure and expertise; limited real-time applicability in acute ICU settings; interpretation complexity [37].

Note: This table summarizes the main diagnostic tools used for the detection and characterization of carbapenem-resistant Enterobacterales (CRE) in intensive care unit (ICU) settings. Each method varies in turnaround time, resolution, and applicability depending on clinical urgency and available resources. **Abbreviations and Acronyms: AST**: Antimicrobial Susceptibility Testing; **CRE**: Carbapenem-Resistant Enterobacterales; **ICU**: Intensive Care Unit; **PCR**: Polymerase Chain Reaction; **WGS**: Whole-Genome Sequencing.

## Data Availability

No new data were created or analyzed in this study. Data sharing is not applicable to this article.
